# The IκB-protein BCL-3 controls Toll-like receptor-induced MAPK activity by promoting TPL-2 degradation in the nucleus

**DOI:** 10.1073/pnas.1900408116

**Published:** 2019-11-26

**Authors:** Patricia E. Collins, Domenico Somma, David Kerrigan, Felicity Herrington, Karen Keeshan, Robert J. B. Nibbs, Ruaidhrí J. Carmody

**Affiliations:** ^a^Centre for Immunobiology, Institute of Infection, Immunity and Inflammation, College of Medicine, Veterinary and Life Sciences, University of Glasgow, Glasgow G12 0YN, United Kingdom;; ^b^Paul O’Gorman Leukaemia Research Centre, Institute of Cancer Sciences, University of Glasgow, Glasgow G12 0YN, United Kingdom

**Keywords:** inflammation, MAPK, nuclear export, proteasomal degradation

## Abstract

Proinflammatory responses induced by Toll-like receptors (TLRs) are dependent on the activation of the NF-ĸB and mitogen-activated protein kinase (MAPK) pathways, which coordinate the transcription and synthesis of proinflammatory cytokines. We demonstrate that BCL-3, a nuclear IĸB protein that regulates NF-ĸB, also controls TLR-induced MAPK activity by regulating the stability of the TPL-2 kinase. TPL-2 is essential for MAPK activation by TLR ligands, and the rapid proteasomal degradation of active TPL-2 is a critical mechanism limiting TLR-induced MAPK activity. We reveal that TPL-2 is a nucleocytoplasmic shuttling protein and identify the nucleus as the primary site for TPL-2 degradation. BCL-3 interacts with TPL-2 and promotes its degradation by promoting its nuclear localization. As a consequence, *Bcl3*^−/−^ macrophages have increased TPL-2 stability following TLR stimulation, leading to increased MAPK activity and MAPK-dependent responses. Moreover, BCL-3–mediated regulation of TPL-2 stability sets the MAPK activation threshold and determines the amount of TLR ligand required to initiate the production of inflammatory cytokines. Thus, the nucleus is a key site in the regulation of TLR-induced MAPK activity. BCL-3 links control of the MAPK and NF-ĸB pathways in the nucleus, and BCL-3–mediated TPL-2 regulation impacts on the cellular decision to initiate proinflammatory cytokine production in response to TLR activation.

## Significance

The NF-ĸB and mitogen-activated protein kinase (MAPK) pathways coordinate the cellular response to most immune stimuli. Toll-like (TLR) and TNF receptor activation of the MAPK pathway requires activation of the TPL-2 kinase. Active TPL-2 is an unstable, short-lived protein, which limits MAPK activity and controls inflammatory responses. Here we report the surprising discovery that active TPL-2 shuttles between the cytoplasm and the nucleus, where it is degraded by the proteasome. BCL-3, a nuclear regulator of NF-ĸB, promotes the nuclear localization and degradation of TPL-2 in order to limit MAPK activity and determines the amount of TLR ligand required to initiate an inflammatory response. Thus, the nucleus is a key site for the integrated regulation of NF-ĸB– and MAPK-driven inflammatory responses.

Toll-like receptor (TLR) activation is essential for effective inflammatory responses to infection or injury. TLR-induced responses are primarily dependent on the activation of the NF-ĸB and mitogen-activated protein kinase (MAPK) pathways (1, 2). Activation of NF-ĸB leads to the transcription of a large number of proinflammatory cytokines, chemokines, and adhesion molecules (3). TLR-induced activation of the MAPK pathway controls the expression of transcription factors such as *Fos*, *Egr1*, and *Elk1* as well as certain cytokines and immunomodulators (2, 4–6). MAPK activation also plays a critical role in the posttranscriptional control of cytokine expression through the regulation of messenger RNA (mRNA) stability, transport, and translation (2). Indeed, NF-ĸB activation in the absence of MAPK activation does not lead to inflammation because increased transcription of cytokine-encoding genes, such as *Tnf*, is not accompanied by mRNA translation (7). Thus, the coordinated control of the NF-ĸB and MAPK pathways is required to ensure that inflammatory responses are appropriately initiated.

Although TLR activation of the NF-ĸB and MAPK pathways both require the IKKβ kinase (8), each pathway possesses distinct features that determine the consequences of TLR ligation. IKKβ-triggered degradation of IĸBα induces the activation of NF-ĸB proportionally to the level of stimulus, thereby leading to a graded transcriptional response (9, 10). In contrast, the MAPK pathway is ultrasensitive; that is, activation in response to increasing concentrations of a stimulus is nonlinear and occurs mostly over a narrow range of ligand concentrations (11, 12). This effectively enables the MAPK pathway to remain insensitive to low levels of stimulus while responding robustly to higher levels (13). Importantly, at TLR ligand concentrations below the MAPK activation threshold, NF-ĸB activation occurs but does not lead to the production of proinflammatory mediators, although, in macrophages at least, it can prime cells for subsequent responses (7, 13).

The serine/threonine kinase TPL-2 (MAP3K8) is essential for MAPK activation by TLR ligands and by cytokines including TNFα and IL1β (14–16). In resting cells, TPL-2 is bound by NF-ĸB p105 which prevents it from interacting with, and phosphorylating, MEK1 (14, 16). Activation of IKKβ phosphorylates TPL-2–bound p105, triggering its ubiquitination and proteasomal degradation. This liberates active TPL-2, which in turn initiates the MAPK cascade by phosphorylating and activating MEK1 (17, 18). Active TPL-2 is highly unstable and is rapidly degraded by the proteasome (14, 16). This is the primary mechanism limiting TLR-induced MAPK activation. Indeed, in the absence of p105 (*Nfkb1*), proteasomal degradation leads to extremely low levels of TPL-2 protein, and *Nfkb1*^−/−^ macrophages and mice are deficient in TLR-induced MAPK activation (16, 17).

Here we show that the IκB protein BCL-3, a known negative regulator of NF-κB transcriptional activity (19), is also an important negative regulator of TLR-induced MAPK activity. We establish TPL-2 as a nucleocytoplasmic shuttling protein and identify an N-terminal nuclear export sequence (NES) in TPL-2 that is sufficient to mediate nuclear export. We demonstrate that TPL-2 undergoes ubiquitination and proteasomal degradation in the nucleus and that nuclear localization is a critical determinant of TPL-2 half-life. BCL-3 binds TPL-2 to promote its proteasomal degradation in the nucleus and thereby regulates MAPK activity. Importantly, BCL-3–mediated inhibition of TPL-2 determines the TLR ligand threshold required to induce proinflammatory cytokine production, and unchallenged *Bcl3*^*−/−*^ mice have elevated levels of circulating proinflammatory cytokines. Together, these findings identify BCL-3 as a unique factor linking the regulation of the MAPK and NF-ĸB pathways in the nucleus that plays a critical role in the cellular decision to initiate production of inflammatory cytokines.

## Results

### BCL-3 Negatively Regulates MAPK-Dependent Gene Expression.

**Fig. 1.**
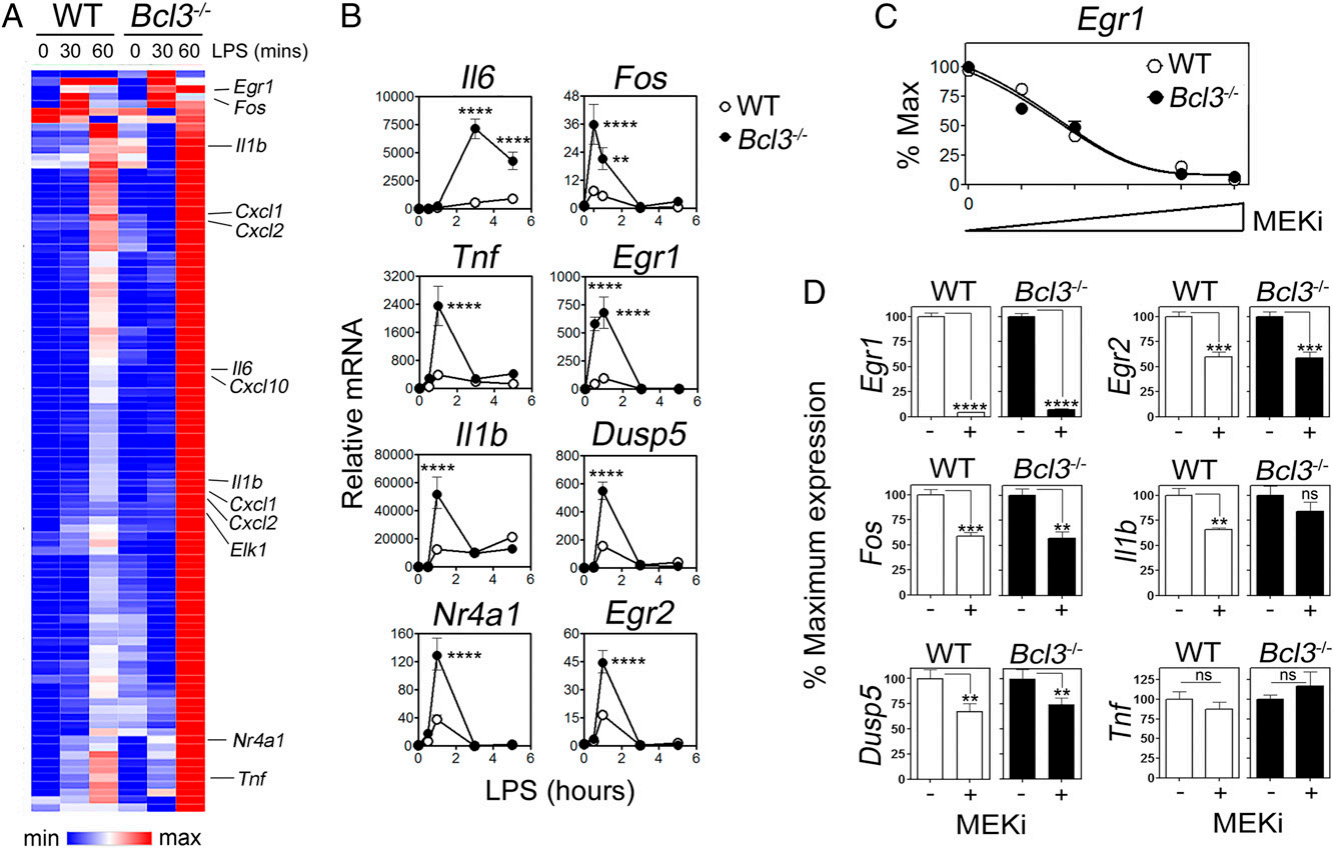
Increased MAPK-dependent gene expression in *Bcl3*^−/−^ BMDMs. (*A*) Heat map of RNA-seq data from WT and *Bcl3*^−/−^ BMDMs stimulated with LPS (10 ng/mL) for the indicated times. Selected genes are indicated. (*B*) qPCR analysis of the expression of selected genes in WT and *Bcl3*^−/−^ BMDMs stimulated with LPS (10 ng/mL) for the indicated times. (*C*) qPCR analysis of *Egr1* expression (10 ng/mL for 60 min) in WT and *Bcl3*^−/−^ BMDMs pretreated for 30 min with increasing amounts (0 to 0.5 µM) of the MEK1 inhibitor U1026 (ME*K*_i_). Relative levels of *Egr1* mRNA are expressed as a percentage (%) of the maximum for each genotype. (*D*) qPCR analysis of LPS-induced expression (10 ng/mL for 60 min) of selected genes in WT and *Bcl3*^−/−^ BMDMs pretreated or untreated with ME*K*_i_ (0.5 µM). Data in *B*–*D* are presented as the mean ± SEM and are representative of 3 independent experiments. Data were analyzed by 2-way ANOVA with the Sidak multiple comparisons test (*B* and *C*) or unpaired Student’s *t* test (*D*). ns, not significant; **P < 0.01; ***P < 0.001; ****P < 0.0001.

We previously identified the IĸB protein BCL-3 as a negative regulator of TLR-induced transcriptional responses through the stabilization of NF-κB p50 homodimer repressor complexes (19, 20). On further analysis we observed that, in addition to inhibiting the expression of NF-κB–regulated proinflammatory genes, BCL-3 also limits the TLR-inducible transcription of a number of immediate/early genes that have previously been shown to rely on MAPK activity for expression (21). Bone marrow-derived macrophages (BMDMs) from wild-type (WT) and *Bcl3*^*−/−*^ mice were left untreated or stimulated with lipopolysaccharides (LPS) (10 ng/mL) and then analyzed by probe-directed RNA-sequencing (RNA-seq) to measure the core TLR-induced transcriptional response. As expected (19), *Bcl3*^*−/−*^ BMDMs were hyper-responsive to LPS stimulation as measured by the elevated expression of NF-κB target genes including *Tnf*, *Il6*, and *Il1b* (Fig. 1*A*). However, LPS-treated *Bcl3*^*−/−*^ BMDMs also showed significantly increased levels of mRNA for the MAPK-dependent genes *Fos*, *Egr1*, *Egr2*, and *Nr4a1* when compared to LPS-treated WT controls (Fig. 1*A* and *SI Appendix*, Table S1). These data were confirmed by qPCR analysis in independent experiments that also demonstrated increased expression of the MAPK-dependent gene *Dusp5* (Fig. 1*B*). Stimulation of BMDMs with other TLR ligands (CpG, PAM3CSK4, or Poly[I:C]) or TNFα also led to increased expression of MAPK target genes in *Bcl3*^−/−^ cells compared to WT cells (*SI Appendix*, Fig. S1).

To confirm the role of MAPK activity (21) in the enhanced expression of these genes, WT and *Bcl3*^*−/−*^ BMDMs were stimulated with LPS (10 ng/mL) in the presence of increasing concentrations of the MEK1 inhibitor U0126 and the expression of *Egr1* measured by qPCR. The LPS-inducible expression of *Egr1* was highly MAPK-dependent in both WT and *Bcl3*^−/−^ cells, which were equally sensitive to MEK1 inhibition (Fig. 1 *C* and *D*). Similar results were seen for LPS-induced expression of *Dusp5*, *Fos*, and *Egr2* while the expression of NF-ĸB–dependent *Tnf* was insensitive to MEK1 inhibition in both WT and *Bcl3*^−/−^ cells (Fig. 1*D*). Together, these data identify a role for BCL-3 in limiting the MAPK-dependent expression of immediate/early genes following TLR stimulation and suggest an NF-κB–independent role for BCL-3 in regulating TLR responses.

### BCL-3 Inhibits TPL-2 Activation of the MAPK Pathway.

**Fig. 2.**
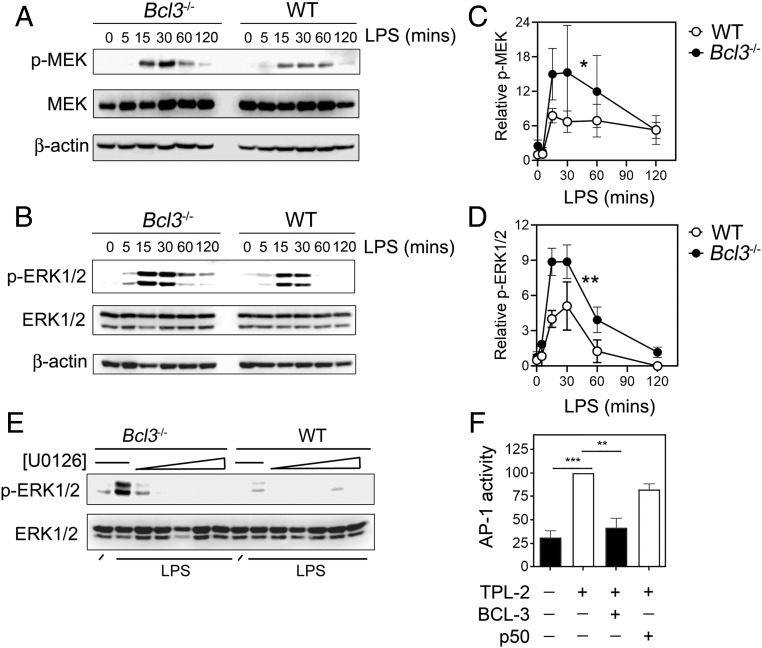
BCL-3 inhibits MAPK activity. WT and *Bcl3*^−/−^ BMDMs were stimulated with LPS (10 ng/mL) for the indicated times, and phosphorylation of MEK1 (*A*) and ERK1/2 (*B*) were analyzed by immunoblot. Phosphorylation of MEK1 (*C*) and ERK1/2 (*D*) were quantified relative to total kinase and β-actin loading controls for 3 independent experiments. (*E*) WT and *Bcl3*^−/−^ BMDMs were untreated or pretreated for 30 min with the MEK1 inhibitor U0126 (0 to 0.5 µM) prior to stimulation with LPS (10 ng/mL for 5 min), and phosphorylation of ERK1/2 was analyzed by immunoblot. (*F*) RAW 264.7 cells were transfected with an AP-1 luciferase reporter plasmid along with expression vectors for TPL-2, BCL-3, or NF-ĸB p50 for 24 h as indicated, and reporter activity was measured. Data are representative of 3 independent experiments. Data in *C*, *D*, and *F* are shown as the mean ± SEM and were analyzed by 2-way ANOVA (*C* and *D*) and 1-way ANOVA (*F*). ***P < 0.001; **P < 0.01; *P < 0.03.

We next analyzed the activation of the MAPK pathway in WT and *Bcl3*^*−/−*^ BMDMs stimulated with LPS (10 ng/mL) using phospho-specific antibodies against MEK1 and ERK1/2. This revealed a significant increase in the LPS-induced activation of the MAPK cascade in *Bcl3*^*−/−*^ cells compared to WT cells, which was characterized by increased phosphorylation of MEK1 and ERK1/2 (Fig. 2 *A*–*D*). LPS-induced activation of the p38 and JNK pathways, as measured by phosphorylation of p38 and c-Jun, was not significantly different between WT and *Bcl3*^−/−^ cells (*SI Appendix*, Fig. S2). The increased LPS-induced activation of ERK1/2 in *Bcl3*^−/−^ cells was sensitive to treatment with MEK1 inhibitor, demonstrating that it results from enhanced MEK1 activity (Fig. 2*E*). TPL-2 (MAP3K8) is the apical kinase of the MAPK pathway activated by TLRs and TNFR, and it directly phosphorylates and activates MEK1. To investigate the effect of BCL-3 on TPL-2–induced MAPK activity independently of TLR activation, we measured the impact of BCL-3 expression on TPL-2–induced AP-1 reporter activity using RAW 264.7 macrophages. Coexpression of BCL-3 and TPL-2 blocked TPL-2–induced AP-1 reporter activity, demonstrating that BCL-3 inhibits TPL-2–induced MAPK activity (Fig. 2*F*). Overexpression of NF-ĸB p50 mimics the effect of BCL-3–mediated inhibition of NF-ĸB target gene expression (19, 20, 22), but this did not block TPL-2–induced AP-1 reporter activity (Fig. 2*F*). Together, these data indicate that BCL-3 can inhibit MAPK activation by TPL-2 through an NF-ĸB–independent pathway.

### BCL-3 Interacts with TPL-2 and Promotes Its Localization to the Nucleus.

**Fig. 3.**
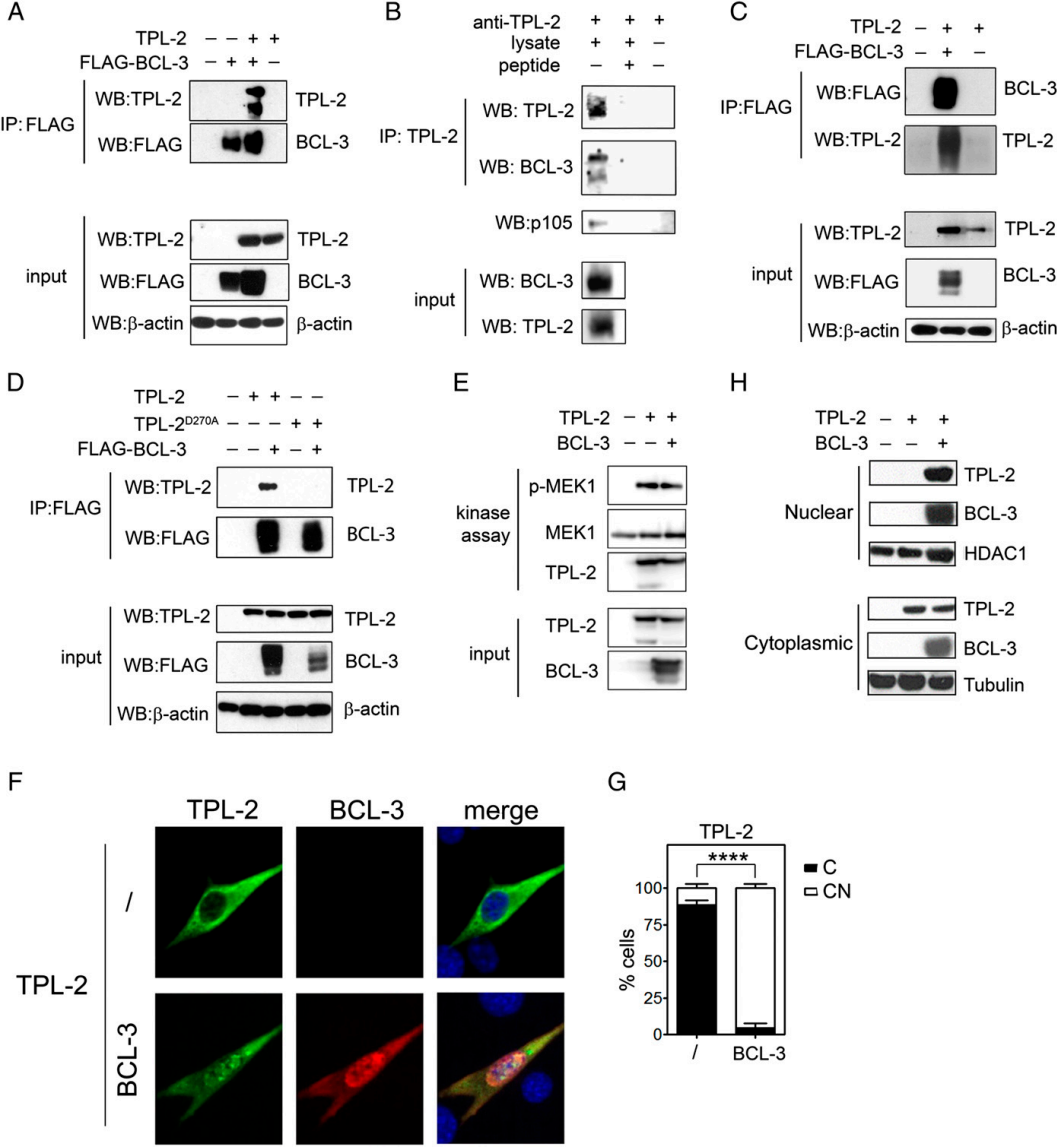
BCL-3 interacts with TPL-2. (*A*) HEK293T cells were transfected for 24 h with plasmids encoding FLAG-BCL-3 and TPL-2 prior to immunoprecipitation of FLAG-BCL-3 and immunoblot using anti–TPL-2 antibody. (*B*) TPL-2 was immunoprecipitated from THP-1 lysates and immunoblotted using anti–BCL-3 antibody. Inhibition of coimmunoprecipitation by the immunizing TPL-2 peptide (peptide) demonstrated antibody specificity. (*C*) *Nfkb1*^−/−^ MEFs were transfected with plasmids encoding FLAG-BCL-3 and TPL-2 for 24 h. Cells were pretreated with 20 µM MG132 for 2 h prior to analysis. Immunoprecipitated FLAG-BCL-3 was immunoblotted with anti–TPL-2 antibody. (*D*) HEK293T cells were transfected with plasmids encoding FLAG-BCL-3, TPL-2, and a kinase dead mutant of TPL-2 (D270A) for 24 h prior to immunoprecipitation of FLAG-BCL-3 and immunoblot using anti–TPL-2 antibody. (*E*) TPL-2 in vitro kinase assay in the presence or absence of recombinant BCL-3, using MEK1 as a substrate. Phosphorylation of MEK1 was detected by anti–p-MEK1 immunoblot. (*F*) Representative confocal immunofluorescence micrograph of 3T3 cells transfected with plasmids encoding TPL-2 and BCL-3 (indicated at the left) and immunostained for TPL-2 and BCL-3 (indicated at the top). DAPI nuclear stain is shown in blue. (Magnification: 40×.) (*G*) Quantification of TPL-2 subcellular localization. The subcellular distribution of TPL-2 was scored as nuclear and cytoplasmic (CN) or predominantly cytoplasmic (C) and presented as the percentage of total cells counted. Data are presented as the mean ± SEM of 3 independent experiments (*n* ≥ 50 cells analyzed per experiment). (*H*) Nuclear and cytoplasmic fractions of HEK293T cells transfected with plasmids encoding TPL-2 and BCL-3 for 24 h were analyzed by immunoblot using the indicated antibodies. All data are representative of at least 3 independent experiments. Data in *G* were analyzed by Student’s *t* test; ****P < 0.0001.

TLR-induced activation of the MAPK and NF-ĸB pathways is linked via NF-ĸB p105, which acts as an inhibitor of both pathways (23). p105 inhibits MAPK activation by binding to TPL-2 through its C-terminal ankyrin repeat domain (16). This domain bears significant structural and sequence homology with the central ankyrin repeat domain of BCL-3, suggesting a potential physical interaction between BCL-3 and TPL-2. TPL-2 protein is expressed as 2 isoforms (58 and 52 kDa) due to alternative translation initiation sites of both endogenous and overexpressed protein (24). Coimmunoprecipitation experiments using HEK293T cells transiently transfected with plasmids encoding BCL-3 and TPL-2 revealed that BCL-3 and TPL-2 can interact in cells (Fig. 3*A*), while coimmunoprecipitation experiments using THP-1 monocytes demonstrated the interaction of endogenous BCL-3 and TPL-2 (Fig. 3*B*). NF-ĸB p105 and p50 are not required for the interaction of BCL-3 and TPL-2 since BCL-3 and TPL-2 also coimmunoprecipitated when expressed in *Nfkb1*^−/−^ mouse embryonic fibroblasts (MEFs) (Fig. 3*C*). Additional experiments using a kinase-inactive mutant of TPL-2 (D270A) revealed that TPL-2 kinase activity is needed for interaction with BCL-3, a requirement similar to that reported for the interaction of TPL-2 with p105 (24) (Fig. 3*D*). These data raise the possibility that the interaction of BCL-3 with TPL-2 inhibits TPL-2 kinase activity to limit the activation of the MAPK pathway and the subsequent transcriptional response to TLR activation. However, an in vitro kinase assay using recombinant MEK1 as substrate revealed that BCL-3 does not inhibit TPL-2–mediated phosphorylation of MEK1 (Fig. 3*E*). Thus, BCL-3 is not a direct inhibitor of TPL-2 kinase activity.

Since it is thought that TPL-2 is a cytoplasmic protein, while BCL-3 is localized to the nucleus, we were interested in determining the subcellular localization of the TPL-2/BCL-3 interaction. This was done using immunofluorescence microscopy and immunoblot analysis of nuclear and cytoplasmic extracts. As predicted, when TPL-2 was expressed alone, it was predominantly found in the cytoplasm (Fig. 3 *F*–*H*). Unexpectedly, however, when coexpressed with BCL-3, TPL-2 showed a striking redistribution from the cytoplasm to the nucleus where it colocalized with BCL-3 (Fig. 3 *F*–*H*). Collectively, these data identify BCL-3 and TPL-2 as interacting partners and reveal that BCL-3 can drive the redistribution of TPL-2 from the cytoplasm to the nucleus. The data demonstrate that BCL-3 and TPL-2 interaction occurs in the nucleus; however, the potential for interaction in the cytoplasm cannot be ruled out.

### TPL-2 Is a Nucleocytoplasmic Shuttling Protein.

**Fig. 4.**
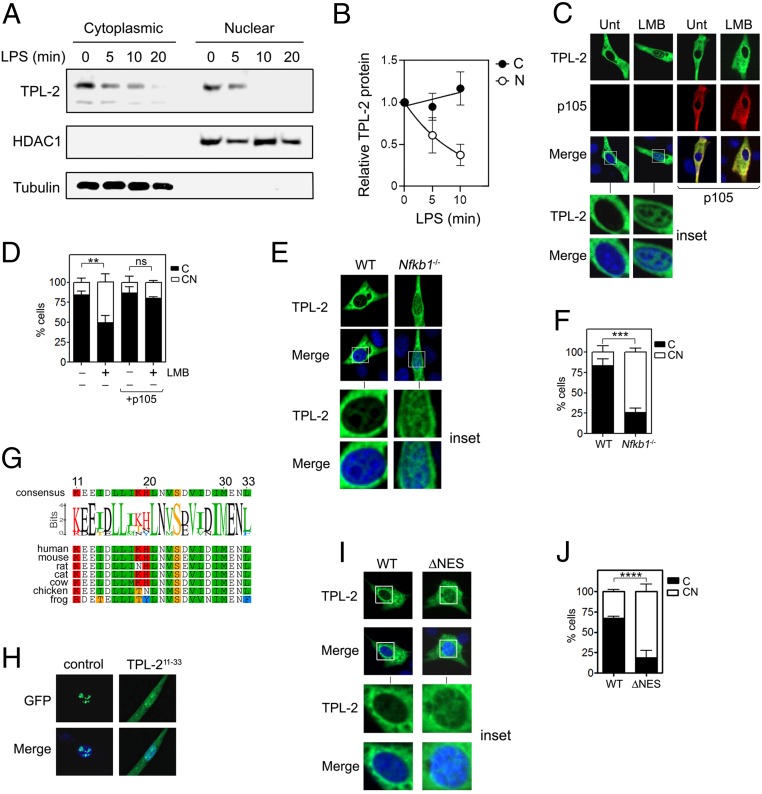
TPL-2 is a nuclear cytoplasmic shuttling protein. (*A*) WT BMDMs were stimulated with LPS (10 ng/mL) for the indicated times before equal amounts of nuclear and cytoplasmic protein were analyzed by immunoblot using the indicated antibodies. (*B*) Quantification of TPL-2 protein levels in cytoplasmic (C) and nuclear (N) fractions relative to loading controls for 3 independent experiments. (*C*) Representative confocal immunofluorescence micrographs of 3T3 cells transfected for 24 h with plasmids encoding TPL-2 and p105 and either untreated (Unt) or treated with leptomycin B (LMB) (20 nM for 2 h). DAPI nuclear stain is shown in blue. (Magnification: 40×.) (*D*) Percentage (%) of cells from (*C*) displaying cytoplasmic (C) or nuclear and cytoplasmic (CN) distribution of TPL-2. (*E*) Representative confocal immunofluorescence micrograph of *Nfkb1*^−/−^ MEFs cells transfected with plasmid encoding TPL-2 for 24 h. DAPI nuclear stain is shown in blue. (Magnification: 40×.) (*F*) Percentage (%) of cells from (*E*) displaying cytoplasmic (C) or nuclear and cytoplasmic (CN) distribution of TPL-2. (*G*) Identification of a conserved NES in the N terminus of TPL-2. (*H*) The TPL-2 NES sequence is sufficient to mediate the nuclear export of a REV-GFP fusion protein. DAPI nuclear stain is shown in blue. (Magnification: 40×.) (*I*) Immunofluorescence microscopy of 3T3 cells transfected for 24 h with plasmid encoding TPL-2 or a TPL-2 mutant lacking the NES (ΔNES). DAPI nuclear stain is shown in blue. (Magnification: 40×.) (*J*) Percentage (%) of cells from *I* displaying cytoplasmic (C) or nuclear and cytoplasmic (CN) distribution of TPL-2. All data are representative of at least 3 independent experiments. Data in *D*, *F*, and *J* are shown as the mean ± SEM of 3 independent experiments (*n* ≥ 50 cells per experiment) and were analyzed by Student’s *t* test. Data in *B* were analyzed by 2-way ANOVA. ns, not significant; **P < 0.01; ***P < 0.001; ****P < 0.0001.

Next, we considered the mechanisms and consequences of TPL-2 localization to the nucleus. Subcellular fractionation demonstrated a readily detectable pool of TPL-2 protein in the nuclear fraction of untreated WT BMDMs (Fig. 4*A*). Both cytoplasmic and nuclear levels of TPL-2 protein rapidly decreased following stimulation with LPS, consistent with the rapid proteasomal degradation of active TPL-2. Of note, after LPS stimulation, TPL-2 protein disappeared more rapidly from the nuclear fraction than from the cytoplasmic fraction, suggesting that it might be more unstable in the nucleus (Fig. 4 *A* and *B*).

To explore the mechanisms underlying the nuclear localization of TPL-2, we treated cells expressing TPL-2 with leptomycin-B (LMB), an inhibitor of Crm1/Exportin-1–mediated nuclear export. This led to a striking accumulation of TPL-2 in the nucleus and strongly suggests that TPL-2 is a nucleocytoplasmic shuttling protein (Fig. 4 *C* and *D*). Interestingly, coexpression of p105 prevented LMB-induced nuclear localization of TPL-2 (Fig. 4 *C* and *D*), and in cells lacking p105, TPL-2 constitutively localized to the nucleus without the requirement for inhibition of Crm1/Exportin-1 (Fig. 4 *E* and *F*). Thus, these data indicate that TPL-2 is a nucleocytoplasmic shuttling protein that can be sequestered in the cytoplasm by p105. These findings are supported by a recent proteomic analysis of Crm1-mediated nuclear export which identified TPL-2 as a Crm1 cargo along with more than 1,000 other proteins (25).

Analysis of the TPL-2 amino acid sequence identified a putative conserved N-terminal NES that is predicted to mediate LMB-sensitive Crm1/Exportin-1–mediated nuclear export (26, 27) (Fig. 4*G*). Insertion of this putative NES into a NES-deficient Rev-GFP fusion protein (28) was sufficient to export the Rev-GFP protein from the nucleus to the cytoplasm, establishing this sequence of TPL-2 as a bone fide NES (Fig. 4*H*). Moreover, deletion of the NES led to the accumulation of TPL-2 in the nucleus (Fig. 4 *I* and *J*) to levels similar to that induced by LMB treatment (Fig. 4*C*) but did not inhibit interaction with p105 or BCL-3 (*SI Appendix*, Fig. S3). These data establish TPL-2 as a nucleocytoplasmic shuttling protein containing an N-terminal NES.

### TPL-2 Undergoes Ubiquitination and Proteasomal Degradation in the Nucleus.

**Fig. 5.**
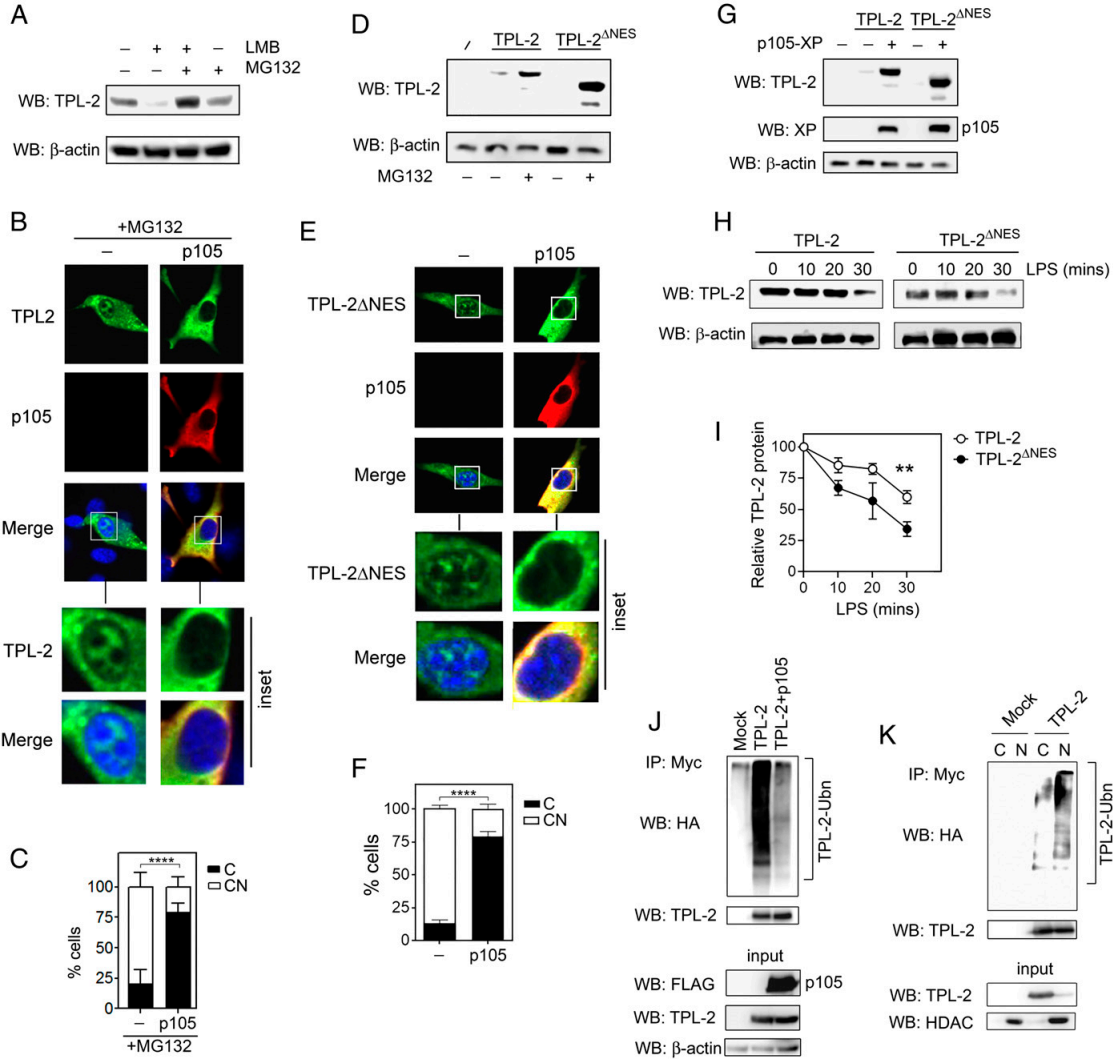
TPL-2 is degraded in the nucleus. (*A*) HEK293T cells transfected for 24 h with plasmid encoding TPL-2 were treated with LMB (20 nM for 6 h) and/or MG132 (20 µM) and analyzed by immunoblot using the indicated antibodies. (*B*) Representative confocal immunofluorescence micrograph of 3T3 cells transfected for 24 h with plasmids encoding TPL-2 and p105 and treated with MG132 (20 µM for 2 h). DAPI nuclear stain is shown in blue. (Magnification: 40×.) (*C*) Percentage (%) of cells from *B* displaying cytoplasmic or nuclear and cytoplasmic distribution of TPL-2. The subcellular distribution of TPL-2 was scored as nuclear and cytoplasmic (CN) or predominantly cytoplasmic (C) and presented as the percentage of total cells counted. Data are presented as the mean ± SEM of 3 independent experiments (*n* ≥ 50 cells per experiment). (*D*) HEK293T cells were transfected for 24 h with plasmids encoding TPL-2 or TPL-2ΔNES and treated with MG132 (20 µM, 4 h) before analysis by immunoblot using the indicated antibodies. (*E*) Representative confocal immunofluorescence micrograph of 3T3 cells transfected with plasmids encoding TPL-2ΔNES and p105 for 24 h. DAPI nuclear stain is shown in blue. (Magnification: 40×.) (*F*) Percentage (%) of cells from *E* displaying cytoplasmic (C) or nuclear and cytoplasmic (CN) distribution of TPL-2ΔNES. Data are presented as the mean ± SEM of 3 independent experiments (*n* ≥ 50 cells per experiment). (*G*) HEK293T cells transfected with plasmids encoding TPL-2, TPL-2ΔNES, and XP-p105 for 24 h and analyzed by immunoblot using the indicated antibodies. (*H*) *Tpl2*^−/−^ Hoxb8-immortalized murine progenitor cells were retrovirally transduced with TPL-2 or TPL-2ΔNES prior to differentiation into macrophages, stimulation with LPS (10 ng/mL) for the indicated times, and immunoblotting with the indicated antibodies. (*I*) TPL-2 protein levels relative to loading controls and normalized to the appropriate untreated control (0 min). (*J*) HEK293T cells were transfected with plasmids encoding Myc-TPL-2, FLAG-p105, and HA-tagged ubiquitin for 24 h. Lysates were immunoprecipitated with antibody against TPL-2 and immunoblotted with antibody against HA. (*K*) HEK293T cells were transfected with plasmids encoding Myc-TPL-2 and HA-tagged ubiquitin for 24 h. Nuclear (N) and cytoplasmic (C) fractions were immunoprecipitated with anti–TPL-2 antibody and immunoblotted with antibody against HA. All data are representative of at least 3 independent experiments. Data in *C* and *F* were analyzed by Student’s *t* test. Data in *I* were analyzed by 2-way ANOVA. **P < 0.01; ****P < 0.0001.

In addition to inducing the nuclear accumulation of TPL-2, LMB treatment also led to a dramatic reduction in cellular TPL-2 protein levels that could be restored by treatment with the proteasome inhibitor MG132 (Fig. 5*A*). These data indicate that TPL-2 may be much more sensitive to proteasomal degradation when it is in the nucleus. This is supported by the finding that, similar to LMB treatment, inhibition of the proteasome led to the nuclear accumulation of TPL-2 which could be reversed by coexpression of p105 (Fig. 5 *B* and *C*). Moreover, deletion of the NES of TPL-2 (TPL-2^ΔNES^) led to reduced TPL-2 levels in cells that could be restored by proteasome inhibition (Fig. 5*D*). p105 expression blocked the nuclear accumulation of TPL-2^ΔNES^ protein (Fig. 5 *E* and *F*) and, like proteasome inhibition, stabilized both full-length TPL-2 and TPL-2^ΔNES^ protein levels (Fig. 5*G*). Hoxb8-immortalized murine myeloid progenitor cells in which *Tpl2* was knocked out using CRISPR/Cas9 (*SI Appendix*, Fig. S4) were reconstituted with TPL-2 or TPL-2^ΔNES^ using retroviral transduction prior to in vitro differentiation to macrophages (*SI Appendix*, Fig. S5). Consistent with transient transfection experiments, TPL-2^ΔNES^ protein levels were reduced compared to full-length TPL-2 (*SI Appendix*, Fig. S5). LPS stimulation induced the degradation of both full-length TPL-2 and TPL-2^ΔNES^ protein levels; however, the degradation of TPL-2^ΔNES^ protein levels occurred more rapidly compared to full-length TPL-2 (Fig. 5 *H* and *I*). An in-cell ubiquitination assay revealed that TPL-2 is highly polyubiquitinated and that this can be inhibited by p105 (Fig. 5*J*). Importantly, the vast majority of polyubiquitinated TPL-2 is detected in the nuclear fraction of cellular extracts (Fig. 5*K*). Collectively, these data identify the nucleus as the site of TPL-2 polyubiquitination and proteasomal degradation and reveal cytoplasmic sequestration of TPL-2 as a key mechanism for the stabilization of TPL-2 by p105.

### BCL-3 Controls TPL-2 Degradation by the Proteasome.

**Fig. 6.**
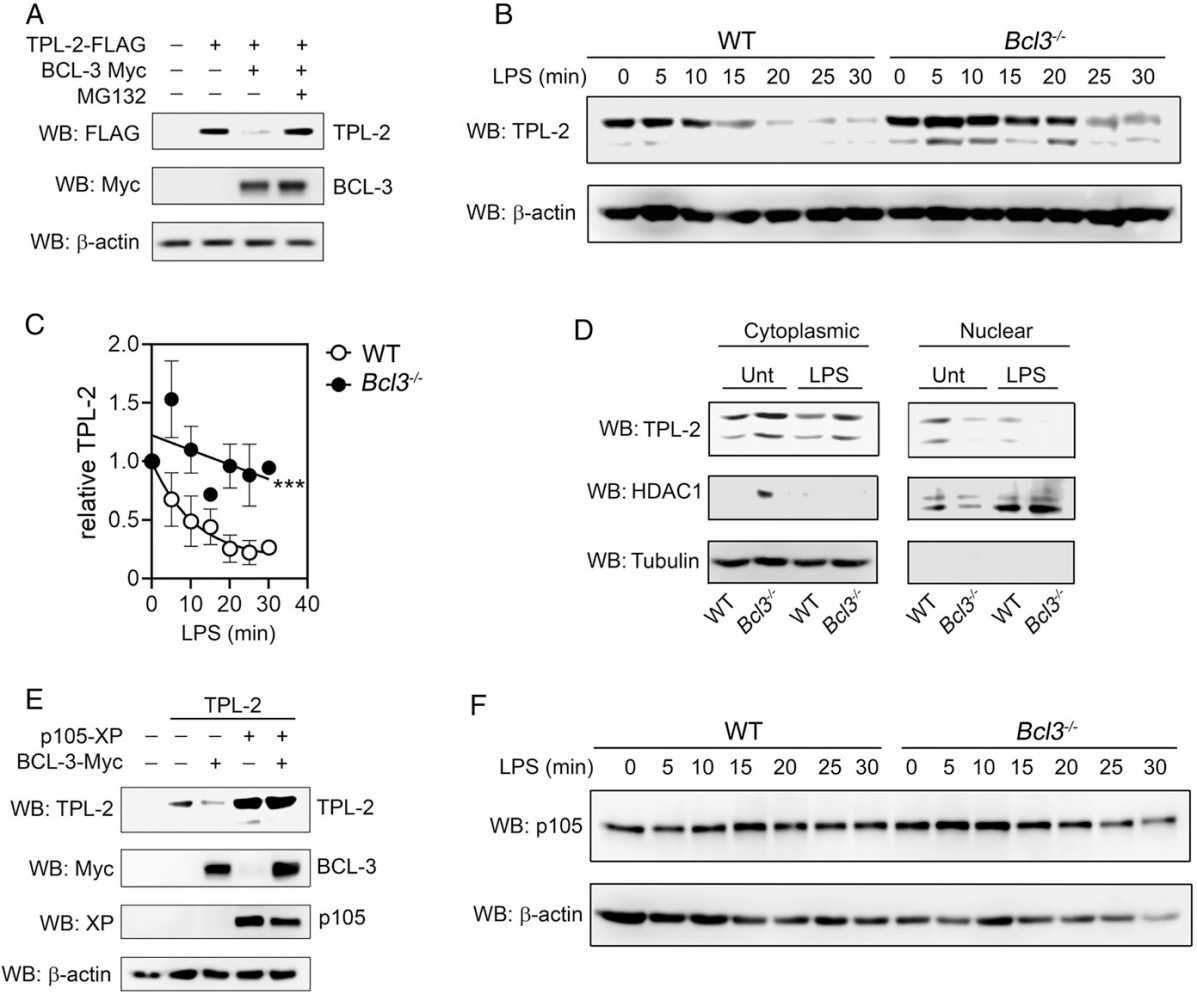
BCL-3 promotes the degradation of TPL-2. (*A*) HEK293T cells were transfected with plasmids encoding Myc-BCL-3 and FLAG-TPL-2 for 16 h and treated with MG132 (20 µM for 4 h) as indicated. Relative protein levels were determined by immunoblotting using the indicated antibodies. (*B*) WT and *Bcl3*^−/−^ BMDMs were stimulated with LPS (10 ng/mL) for the indicated times prior to analysis by immunoblotting using the indicated antibodies. (*C*) TPL-2 levels were quantified relative to loading controls for 3 independent experiments as shown in *B*. (*D*) WT and *Bcl3*^−/−^ BMDMs were untreated (Unt) or stimulated with LPS (10 ng/mL for 10 min) before nuclear and cytoplasmic fractions were analyzed by immunoblotting using anti–TPL-2 antibody. (*E*) HEK293T cells were transfected for 16 h with plasmids encoding TPL-2, XP-p105, and Myc-BCL-3 as indicated. Relative protein levels were determined by immunoblotting. (*F*) WT and *Bcl3*^−/−^ BMDMs were stimulated with LPS (10 ng/mL) for the indicated times prior to analysis by immunoblotting using the indicated antibodies. All data are representative of at least 3 independent experiments. *P* < 0.0005.

In light of these findings, we predicted that BCL-3–induced nuclear localization of TPL-2 would lead to TPL-2 degradation and provide an explanation for the ability of BCL-3 to inhibit MAPK activity (Figs. 1 and 2). Indeed, expression of BCL-3 induced a dramatic reduction in TPL-2 protein levels that was reversed by proteasome inhibition (Fig. 6*A*). Moreover, the half-life of TPL-2 in LPS-stimulated *Bcl3*^−/−^ BMDMs was ∼2-fold greater than that of WT BMDMs (Fig. 6 *B* and *C*). In addition, compared to WT cells, *Bcl3*^−/−^ BMDMs contained significantly less nuclear, and more cytoplasmic, TPL-2 in both the presence and the absence of LPS stimulation (Fig. 6*D*). As expected from the data above (Fig. 5), the expression of p105 prevented BCL-3–induced degradation of TPL-2 (Fig. 6*E*). There were equivalent levels of p105 expression in LPS-stimulated *Bcl3^−/−^* cells compared to WT cells (Fig. 6*F*) consistent with our previous findings (19). Thus, by shifting the equilibrium of TPL-2 nucleocytoplasmic shuttling toward the nucleus, BCL-3 increases the proteasomal degradation of TPL-2 to limit MAPK activation.

### BCL-3 Sets the Activation Threshold for LPS-Induced Responses.

**Fig. 7.**
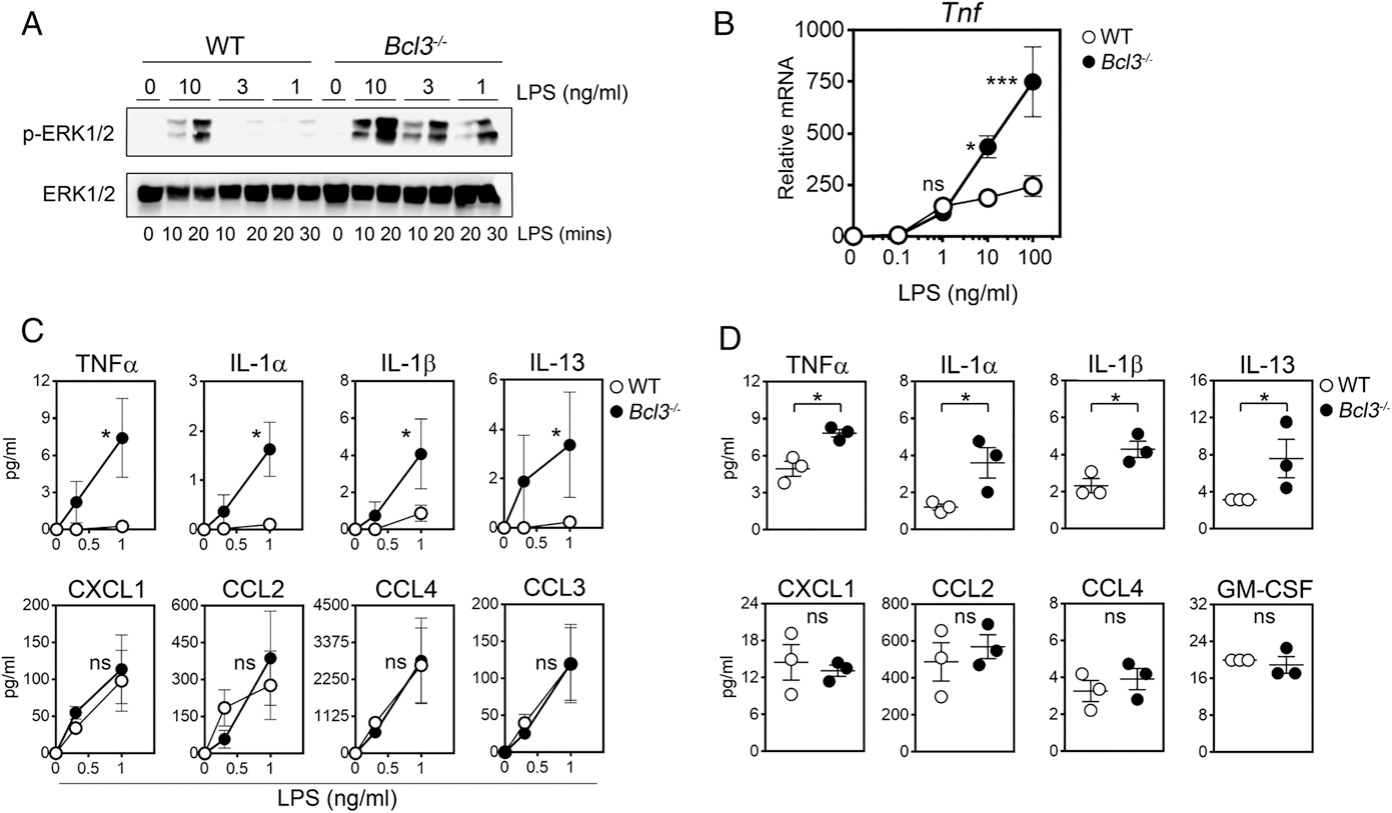
BCL-3 regulates the MAPK response threshold to TLR activation. (*A*) WT and *Bcl3*^−/−^ BMDMs were stimulated with the indicated concentration of LPS, and phospho-ERK1/2 levels were measured by immunoblotting at the indicated time points. Data are representative of 3 independent experiments. (*B*) WT and *Bcl3*^−/−^ BMDMs were stimulated with the indicated concentrations of LPS, and *Tnf* mRNA levels were measured by qPCR. Data are presented as the mean ± SEM and are representative of 3 independent experiments. (*C*) WT and *Bcl3*^−/−^ BMDMs were stimulated with the indicated concentrations of LPS, and levels of secreted cytokines and chemokines were measured. The means ± SEM of 3 independent experiments are shown. (*D*) Serum from WT and *Bcl3*^−/−^ mice was analyzed for the indicated factors. The means ± SEM are presented. Data were analyzed by 2-way ANOVA with Sidak multiple comparisons test (*B* and *C*) or Student’s *t* test (*D*). ns, not significant; *P < 0.05; ***P < 0.001.

BCL-3 regulation of the MAPK pathway clearly suppresses the expression of certain genes induced by 10 ng/mL LPS (Fig. 1) and will co-operate with its regulation of NF-ĸB activity to dictate the magnitude and duration of transcriptional responses induced by medium-to-high quantities of TLR ligand (19). We were also interested in whether BCL-3 regulates the set point of the MAPK activation threshold. In WT cells this limits MAPK activation at low TLR ligand concentrations and means that the MAPK pathway, unlike the NF-κB pathway, is not activated in a linear fashion (13). Thus, we first measured MAPK activity in WT and *Bcl3*^*−/−*^ BMDMs stimulated with a range of LPS concentrations (1 to 10 ng/mL). Strikingly, the MAPK pathway was activated at significantly lower concentrations of LPS in *Bcl3*^−/−^ BMDMs compared to WT BMDMs (Fig. 7*A*). As expected (19, 20), the activation of the NF-ĸB pathway, as measured by IĸBα degradation, was equivalent between WT and *Bcl3*^−/−^ cells (*SI Appendix*, Fig. S6).

To assess the impact of this altered MAPK activation threshold in *Bcl3*^−/−^ macrophages, we next analyzed LPS-induced production of cytokines and chemokines. At 10 to 100 ng/mL of LPS, *Tnf* mRNA was significantly higher in *Bcl3*^−/−^ BMDMs due to loss of BCL-3–mediated regulation of the NF-ĸB pathway. At the low levels of LPS (1 ng/mL) that induced MAPK activity in *Bcl3*^*−/−*^, but not WT cells, there were no significant differences in *Tnf* mRNA between WT and *Bcl3*^*−/−*^ macrophages (Fig. 7*B*). However, TLR-induced production of TNFα protein is known to rely on MAPK-dependent posttranscriptional mechanisms controlled by TPL-2 activation (4, 6). Thus, we also examined cytokine release from LPS-treated BMDMs. Strikingly, only *Bcl3*^*−/−*^ BMDMs released TNFα in response to stimulation with the low doses of LPS that activated the MAPK pathway only in *Bcl3*^*−/−*^ BMDMs (Fig. 7*C*). Similarly, low-dose LPS stimulation resulted in the production of the IL-1α, IL-1β, and IL-13 cytokines by *Bcl3*^*−/−*^, but not WT, BMDMs (Fig. 7*C*). This effect was selectively associated with proinflammatory cytokines because the production of the CXCL1, CCL2, CCL3, and CCL4 chemokines, which relies primarily on the NF-ĸB pathway (7), was equivalent between WT and *Bcl3*^*−/−*^ cells treated with the same low concentrations of LPS (Fig. 7*C*). Thus, BCL-3 controls the MAPK activation threshold to prevent the production of proinflammatory cytokines when cells are exposed to very low levels of LPS.

As expected (19), the serum of *Bcl3*^−/−^ mice injected with LPS (2.5 µg) contained more cytokines and chemokines than similarly treated WT animals (*SI Appendix*, Fig. S7). However, in unchallenged mice, serum concentrations of TNFα, IL-13, IL-1α, and IL-β were all found to be significantly higher in *Bcl3*^−/−^ mice than in WT counterparts, while the concentrations of the chemokines CXCL1, CCL2, and CCL4 and GM-CSF were not different (Fig. 7*D*). These data mirror those seen with BMDMs to suggest that BCL-3, by regulating the MAPK activation threshold, sets the baseline level of proinflammatory cytokine production in vivo.

## Discussion

This study identifies a role for the IĸB protein BCL-3 in limiting inflammatory responses to TLR activation independently of its previously described role in regulating NF-ĸB transcriptional activity (19). It establishes BCL-3 as a unique factor that integrates the control of the NF-ĸB and MAPK pathways in the nucleus and, furthermore, identifies the nucleus as a key site for the regulation of TLR-induced MAPK activity.

The proteasomal degradation of TPL-2 has long been recognized as a key factor in limiting TLR- and cytokine-induced MAPK activity (16). However, apart from the stabilization of TPL-2 by p105 (14, 16), relatively little is known about the mechanisms controlling this process. The compartmentalization of TPL-2 ubiquitination and proteasomal degradation to the nucleus indicates that factors that influence TPL-2 subcellular localization will have a profound impact on MAPK activity. Thus, the opposing effects of BCL-3 and p105 on TPL-2 nuclear localization are mirrored by significant changes in TPL-2 stability and TLR-induced MAPK activity. The mechanisms underlying TPL-2 nuclear localization are presently unclear. Although sequence analysis of TPL-2 does not reveal an obvious nuclear localization sequence, this does not rule out that TPL-2 may possess intrinsic nuclear localization properties. The presence of TPL-2 in the nucleus of *Bcl3*^−/−^ cells suggests that BCL-3 is not required for the nuclear import of TPL-2; however, it is possible that another factor(s) may play a role in TPL-2 nuclear import. It is interesting to note that the interaction of TPL-2 with 14–3-3 has previously been reported to promote TPL-2–mediated activation of the MAPK pathway (29). Previous studies have shown that 14–3-3 also acts as a molecular chaperone to sequester a number of proteins in the cytoplasm, including IĸBα:p65 complexes (30), HDACs (31), and CDC25 (32). It remains to be seen if the sequestration of TPL-2 in the cytoplasm contributes to 14–3-3–mediated activation of the MAPK pathway. The dependence of TPL-2 activation on proteasomal degradation of p105 excludes the use of proteasome inhibitors for the investigation of TPL-2 degradation in the nucleus. The identification of the E3 ligase complex responsible for TPL-2 ubiquitination will be important to further define the mechanism controlling TPL-2 degradation and will likely reveal additional checkpoints controlling TLR responses that could be targeted for the therapeutic modulation of inflammatory responses.

The essential requirement of MAPK activation for the initiation of inflammation is perhaps most clearly demonstrated in studies of transgenic mice with constitutive activation of NF-ĸB in intestinal epithelial cells (7). These cells show elevated expression of NF-ĸB target genes including cytokines, such as *Tnf*, and chemokines, such as *Ccl2*, but in the absence of MAPK activation the production of TNFα protein was too low to induce inflammation. Strikingly, the mechanisms of MAPK-dependent posttranscriptional control did not apply to the production of chemokines which were abundantly expressed in response to NF-ĸB activation alone. The result was leukocyte infiltration of the lamina propria in the absence of inflammatory tissue damage (7). Thus, the absence of MAPK activation uncoupled the activation of NF-ĸB from inflammation. Likewise, the MAPK activation threshold will determine the precise set point of when a cell responds to TLR activation irrespective of the NF-ĸB pathway. The lower MAPK activation threshold in *Bcl3*^−/−^ cells reveals that the TPL-2 half-life is a critical determinant in setting the TLR ligand concentrations required to initiate inflammation. The selective dependence of proinflammatory cytokine, but not chemokine, production on MAPK activation is reflected in the patterns of cytokine and chemokine expression at subthreshold levels of the TLR ligand in the absence of BCL-3 in vitro and in vivo.

This study establishes BCL-3 as a unique factor that regulates the MAPK activation by modifying the stability of an active apical kinase. It suggests that the modulation of BCL-3 expression by cytokines such as IL-10 (33) may also shift the MAPK threshold and thereby raise the concentration of ligand required to initiate inflammation through TLRs. It also identifies BCL-3 as a target for modulating inflammatory responses by altering the sensitivity of innate cells to TLR stimulation. Modulating the stability or nuclear export of TPL-2 would have a similar effect; for example, reduced TPL-2 nuclear export would increase the MAPK activation threshold and the concentration of TLR ligand or cytokine required to initiate an inflammatory response. The potential to modulate the MAPK activation threshold is an attractive strategy for limiting inflammatory responses without preventing the capacity of cells to initiate inflammation when a clear threat is encountered. It is important to further understand mechanisms controlling the MAPK activation threshold so that this potential therapeutic approach can be successfully exploited.

The dual function of BCL-3 as an inhibitor of both NF-ĸB transcriptional activity and MAPK activity establish it as a key factor integrating control of TLR responses in the nucleus. The involvement of the ubiquitin proteasome system in BCL-3–mediated control of both pathways further underlines the importance of this cellular mechanism in the control of inflammatory responses. However, the importance of TPL-2 nuclear localization in the control of MAPK responses was unexpected and challenges the accepted models of signal transduction that lead from receptor proximal events in the cytoplasm, through incremental stages, to the activation of transcription factors in the nucleus. Instead, this study indicates that the dynamic movement of key upstream activators of signaling pathways between the cytoplasm and the nucleus can play a critical role in determining the outcome of receptor activation. The potential involvement of similar mechanisms in the control of other immunoreceptor-mediated responses requires investigation.

In summary, this study identifies the nuclear cytoplasmic shuttling of TPL-2 as a mechanism regulating TLR-induced MAPK activity and the MAPK activation threshold. Our data establish BCL-3 as a unique factor that integrates the regulation of MAPK activation and NF-ĸB activity in the nucleus. The possibility of modifying MAPK activity, and the MAPK activation threshold, through altering TPL-2 localization, stability, or interaction with BCL-3 offers the potential to modify inflammatory responses for therapeutic benefit.

## Materials and Methods

### Cell Culture, CRISPR/Cas9 Gene Editing, and Retroviral Transduction.

HEK293T and RAW264.7 cells were cultured in Dulbecco’s Modified Eagle Media (DMEM) containing 10% fetal bovine serum (FBS), and 3T3s MEFs in DMEM containing bovine calf serum with 2 mM glutamine and 100 units/mL penicillin/streptomycin. THP-1 cells were cultured in RPMI containing 10% FBS with of 2 mM glutamine and 100 units/mL penicillin/streptomycin. BMDMs were prepared in vitro as described previously (34). Hoxb8-immortalized murine myeloid progenitor cells and derived macrophages were generated as previously described and cultured in RPMI supplemented with 10% FBS, GM-CSF (20 ng/mL), and β-estradiol (1 µM) (35). CRISPR/Cas9 gene editing and retroviral transduction are detailed in *SI Appendix*, *Materials and Methods*.

### Plasmids, Transfection, and Reagents.

Mammalian expression vectors for BCL-3 and p105 were as previously described (19). TPL-2-MYC and TPL-2^D270A^- MYC in pcDNA3 were generous gifts from Stephen Ley, Francis Crick Institute, London (14). pcDNA3.1-TPL-2-*N*-FLAG expression vectors were generated by Genscript using TPL-2 cDNA accession number NM_053847. pRevGFP and pRev(1.4)-GFP were generous gifts from Beric Henderson, University of Sydney, Australia, and are described elsewhere (28). Transfection reagents and the cloning of the TPL-2 NES into pREV(1.4)-GFP are detailed in *SI Appendix*, *Materials and Methods*.

### Western Blot and Immunoprecipitation.

Whole-cell or cytoplasmic and nuclear proteins were extracted as previously described (36) and as detailed in *SI Appendix*, *Materials and Methods*.

### Luciferase Assay.

The AP-1 promoter reporter plasmid (Clontech) and the Renilla-luciferase expression vector pRL-TK (Promega) were used as detailed in *SI Appendix*, *Materials and Methods*.

### Gene Expression.

Targeted RNA-seq analysis was performed using the QIA-seq Mouse Inflammation and Immunity Transcriptome-targeted RNA Panel (RMM-005Z) as detailed in *SI Appendix*, *Materials and Methods*.

### Cytokine Analysis.

Cytokine concentrations for supernatants and serum samples were measured using BioRad Bio-plex Pro mouse cytokine Grp1 Panel 23-plex according to the manufacturer’s instructions using the Bio-Plex 200 system as detailed in *SI Appendix*, *Materials and Methods*.

### Immunofluorescence Microscopy.

Cells were fixed and permeabilized with ice-cold methanol for 15 min, blocked in phosphate-buffered saline/0.05% Tween containing 5% bovine serum albumin, and incubated with antibodies as detailed in *SI Appendix*, *Materials and Methods*.

### Kinase Assay.

TPL-2 kinase assays were performed using GST-MEK1 as a substrate as described in *SI Appendix*, *Materials and Methods*.

### Animal Studies.

*Bcl3*^*−/−*^ mice (37) were bred in‐house (C57BL/6 background); WT C57BL6/J mice were from Charles River Research Models and Services. Animal work was carried out with ethical approval from the University of Glasgow under the revised Animal (Scientific Procedures) Act of 1986 and the European Union Directive 2010/63/EU.

### Data Availability.

All data and associated protocols are available in *SI Appendix*. Plasmids and cell lines are available on request.

## Supplementary Material

Supplementary File

Supplementary File
