# Supplementary material for: The IκB-protein BCL-3 controls Toll-like receptor-induced MAPK activity by promoting TPL-2 degradation in the nucleus
Source: Proc Natl Acad Sci U S A. 2019 Nov 26;116(51):25828–38. doi: 10.1073/pnas.1900408116 (PMC6926074; doi:10.1073/pnas.1900408116)
Supplement: Supplementary File [file pnas.1900408116.sapp.pdf]

Supplementary Information Appendix

www.pnas.org/cgi/doi/10.1073/pnas.1900408116

MATERIALS AND METHODS

Cell Culture, CRISPR/Cas9 gene editing and retroviral transduction

Briefly, bone marrow was isolated from age- and sex- matched C57BL6/J mice at 8-12 weeks old, and cultured in bacterial petri dishes with DMEM containing 10% fetal bovine serum, 2mM glutamine, and 100 units/ml penicillin/streptomycin supplemented with 30% L929 conditioned media for 7 days, with media replacement after four days. On day 7, BMDMs were harvested using PBS/5mM EDTA solution and re-plated into tissue culture-treated dishes without supplementation with L929 conditioned media. They were rested overnight and experiments performed on day 8. Hoxb8 immortalised murine myeloid progenitor cells were differentiated to macrophages by washing cells twice in medium without β-estradiol following by incubated in RPMI supplemented with 10% fetal bovine serum and GM-CSF (20ng/ml) for 5 days. CRISPR/Cas9 knockout of Tpl2 was performed by electroporation of sgRNA and recombinant Cas9 (Synthego) using a Neon electroporator (Invitrogen) and single cell clones screened for *Tpl2* knockout (SI Appendix, Fig. S4). *Tpl2* and *Tpl2ΔNES* were cloned into the MSCV PIG retroviral vector (gift from Scott Lowe; Addgene plasmid # 18751) and transduction of *Tpl2*$^{-/-}$ cells performed as previously described (1) and transduced cells were isolated by puromycin selection. Retroviral transduction was verified by flow cytometric analysis of GFP expression. Briefly, cells were incubated with viability dye eFluor 780 (eBioscience) for 30 min at 4 °C to exclude dead cells, and the amount of GFP-labelled cells measured using a BD ARIA III. Data was analysed using BD FACSDiva software 8.0.1 and FlowJo software 10.6.1.

Plasmids, Transfection and reagents

The DNA fragment coding for the putative TPL-2 nuclear export sequence KEEIDLLINHLNVSEVLDIMENLYA was cloned into the pRev (1.4)-GFP vector via BamHI and AgeI sites. The specific oligonucleotides used were as follows : 5’GATCCAAAAGAAGAGATTGATTTATTAATTAACCATTTAAACGTGTCGGAAGTCCTGGA CATCATGGAGAACCTTTATGCAA3’ and 5’CCGGTTGCATAAAGGTTCTCCAGATGTCCAGGACTTCCGACACGTTTAAATGGTTAAT

TAATAAATCAATCTCTTCTTTTG3’ (Integrated DNA Technologies). Cells were transfected for 16-24 hours using the *in vitro* transfection reagents: Turbofect Fermentas (293T), attractene, Qiagen (3T3) and Fugene HD, Promega (RAW264.7) according to manufacturer’s protocol. LPS from Escherichia coli 055:B5, LMB from Streptomyces *sp*, MG132 and U0126 monoethanolate were all purchased from Sigma.

Western blot and immunoprecipitation

For all cell types used, whole cell lysates were prepared from cells previously washed twice in ice-cold PBS and suspended in RIPA buffer containing 50mM Tris-HClI pH7.4, 1% NP-40, 0.25% deoxycholate, 150mM NaCl, 1mM EDTA, supplemented with 1mM PMSF, 1mM NaF, 1mM Na3VO4, 2µg/ml aprotinin, 1µg/ml pepstatin and 1µg/ml leupeptin. Nuclear and cytoplasmic extracts from HEK293T cells were obtained using a Nuclear Extract kit according to manufacturer’s instructions (Active Motif). When performing extractions from BMDM for endogenous nuclear and cytoplasmic TPL-2, the concentrations of kit phosphatase and protease inhibitors were doubled and lysis buffers were additionally supplemented with (aprotinin, pepstatin, leupeptin and MG132). Lysates were resolved using Tris-Glycine SDS-PAGE, transferred to nitrocellulose membranes and immunoblotted with specific antibodies. For co-immunoprecipitation experiments, equal protein concentrations of whole cell extracts from were precleared for 30 minutes at 4°C with protein G agarose beads and immunoprecipitated with primary antibody overnight at 4°C. Agarose pellets were then washed three times in RIPA buffer and eluted by incubation at 95°C in 2X sample buffer (4% SDS, 20% glycerol, 10% 2-mercaptoethanol, 0.004% bromophenol blue and 0.125 M Tris HCl pH6.8). For endogenous immunoprecipitation experiments, RIPA buffer was supplemented with 20µm MG132 and TPL-2 was eluted using the TPL-2 blocking peptide instead of 2x sample buffer by incubation at room temperature for 10 minutes with occasional agitation (Santa Cruz). Equal volumes of resuspended immunoprecipitates were analysed by western blot. For ubiquitination assays, cells were incubated with 10mM N-ethylmaleamide (NEM) for 30 seconds and washed in PBS/10mM NEM. Cells were lysed in 1% SDS, boiled for 5 min

and sonicated. Cleared lysates were diluted (1/10) in RIPA buffer supplemented with 20mM NEM. Immunoprecipitation was performed as above and analysed by western blot.

Cytokine analysis

To determine cytokine concentrations in vitro, $1x10^5$ BMDM were plated per well of a flat bottom 96-well microplate in 200µl of complete media. Cells were left unstimulated or stimulated with 0.1-10ng/ml of LPS for 4 hours. For each individual experiment, supernatants from 3 technical replicates were combined and stored at -80°C until analysis. Serum samples were diluted 1:3 with sample diluent (BioRad). Cytokine concentrations for supernatants and serum samples were measured using BioRad Bio-plex Pro mouse cytokine Grp1 Panel 23-plex according to manufacturer's instructions using the Bio-Plex 200 system.

Antibodies

Anti-MYC (sc-40,) anti-TPL-2 M20 (sc-720) and anti-TPL-2 H7 (sc-373677) were purchased from Santacruz. Anti-FLAG rabbit (SAB 4301135), anti-FLAG M2 mouse (F1804), anti-HDAC1 (AV38530), anti α-Tubulin (t6074) and anti-B-actin (SAB 1305567) were purchased from Sigma. Antibodies against p-ERK (#9103), ERK (#9102), p-MEK (#9154), MEK (#4694), pRSK (#23556) and RSK2 (#9340) were purchased from Cell Signalling Technology and anti-Xpress from Invitrogen. Secondary antibodies for western blotting were purchase from GE healthcare. AF-488 anti-rabbit (A11008), AF-594 anti-mouse (A11005) AF-594 anti-rabbit (A11012) immunofluorescence secondary antibodies were from Life technologies.

Luciferase Assay

$1x10^5$ RAW 264.7 cells were plated per well of a 24 well plate and transiently transfected with pAP-1, an AP-1 promoter reporter plasmid (Clontech) and the Renilla-luciferase expression vector pRL-TK (Promega) in addition to TPL-2, Bcl-3 and p50 expression vectors for 24 hours. Luciferase activity was measured using the Promega's dual-Luciferase reporter assay system. For all samples, firefly luciferase activity was divided by that of the Renilla luciferase activity

to normalize for the transfection efficiency.

Gene expression

$1x10^6$ BMDMs were plated per well of a 12 well plate and total RNA was isolated using RNeasy kits (Qiagen) and reversely transcribed using NanoScript 2 reverse transcription kit (Primer Design). QPCR was performed with SYBR Green SuperMix with ROX (PerfeCTa) using QuantiTect Primer Assays (Qiagen). Data were normalised to TBP and gene expression changes calculated using the $2^{-\Delta\Delta CT}$ method. Targeted RNA-seq analysis was performed using the QIA-seq Mouse Inflammation and Immunity Transcriptome targeted RNA Panel (RMM-005Z) according to the manufacturer's instructions. Data analysis was performed using the Qiagen Data Analysis Center (https://www.qiagen.com/gb/shop/genes-and-pathways/data-analysis-center-overview-page/).

Immunofluorescence microscopy

$0.7x10^6$ WT or *Nfkb1*$^{-/-}$ 3T3s were plated in a 10cm tissue culture dish containing glass coverslips and transfected for 24 hours. For treatments, cells were incubated with vehicle controls or with 20nM LMB (Sigma) and 20µM MG132 (Sigma) alone or in combination for 2 hours. Cells were fixed and permeabilised with ice-cold methanol for 15 minutes, blocked in PBS-0.05% TWEEN (PBS-T) containing 5% BSA. Primary and secondary antibodies were diluted in 1%BSA/PBS-T and staining was performed overnight at 4°C and for 1 hour at room temperature respectively. Nuclear DNA was counterstained using DAPI mounting medium (Vectashield, Vector Laboratories). Immunofluorescent images were acquired using a Zeiss Cell Observer SD confocal fluorescent microscope (Zeiss). The subcellular distribution of TPL-2 was scored as nuclear and cytoplasmic (NC) or predominantly cytoplasmic (C) and presented as the percentage of total cells counted. A total of 50 or more cells were analysed for each transfection and experiments were performed in triplicate.

Kinase assay

For TPL-2 kinase assays, HEK293T cells were transiently transfected with pRK5-Bcl-3-FLAG and pcDNA3.Tpl-2-MYC expression vectors for 24 hours as indicated. TPL-2 was then immunoprecipitated (IP) from whole cell lysates overnight with anti-MYC and the following day an *in vitro* MEK kinase assays (KA) performed with inactive GST-MEK1. Following TPL-2 immunoprecipitation, agarose beads were washed 10 times in 1ml of RIPA buffer and washed twice in 1ml MEK kinase assay buffer (50 mM Tris [pH 7.5], 150 mM NaCl, 5 mM β-glycerophosphate, 2 mM dithiothreitol, 0.1 mM sodium vanadate, 10 mM $MgCl_2$, 1 mM EGTA, 0.01% Brij-35). Beads were then incubated in 20µl of MEK kinase assay buffer containing 1µg recombinant inactive MEK1 20µl (Millipore) and 2mM adenosine triphosphate at 30°C with occasional agitation for 15 minutes. Beads were pelleted at 11,000g, the supernatant removed, added to 20µl 2X SDS sample buffer. MEK phosphorylation was determined by western blot of kinase assay reaction with anti-phospho MEK1/2 antibody. Immunoprecipitates were also eluted from the beads and analysed for equal loading with anti-TPL-2 by Western blot.

Animal studies

Animals were co-housed in individual ventilated cages in a barrier facility proactive in environmental enrichment. *Bcl3*$^{-/-}$ mice (2) were bred in-house (C57BL/6 background); wild type (WT) C57BL6/J mice were from Charles River Research Models and Services. All experimental mice were sex- and gender-matched and used between the ages of 6 and 12 weeks. Animal work was carried out with ethical approval from University of Glasgow under the revised Animal (Scientific Procedures) Act 1986 and the European Union Directive 2010/63/EU. All experiments were performed in accordance with relevant guidelines and regulations. The numbers of animals used in each experiment are noted in the relevant figure legends. LPS (2.5µg/mouse) was administered *i.p* and serum collected from a tail bleed after 1 hour.

1. Chaudhury S*, et al.* (2018) Age-specific biological and molecular profiling distinguishes paediatric from adult acute myeloid leukaemias. *Nat Commun* 9(1):5280.
2. Schwarz EM, Krimpenfort P, Berns A, & Verma IM (1997) Immunological defects in mice with a targeted disruption in Bcl-3. *Genes Dev* 11(2):187-197.

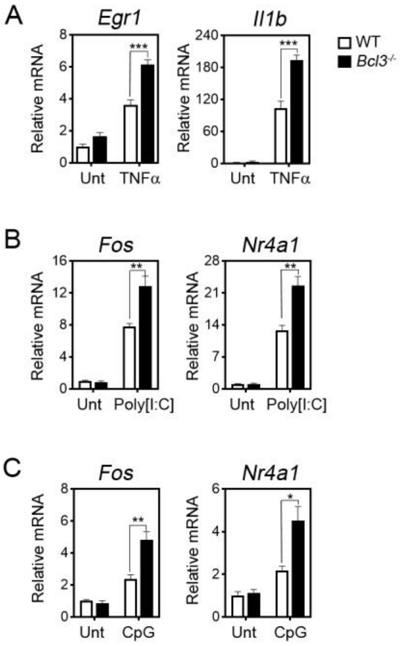

Figure S1 Increased expression of MAPK-regulated genes in *Bcl3*$^{-/-}$ BMDMs treated with TLR ligands or TNFα. (A) WT and *Bcl3*$^{-/-}$ BMDMs were stimulated with TNFα (0.2ng/ml for 60 mins) and *Egr1* and *Il1b* mRNA measured by QPCR. **(B)** WT and *Bcl3*$^{-/-}$ BMDMs were stimulated with Poly[I:C] (50µg/ml for 60 mins) and *Fos* and *Nr4a1* mRNA measured by QPCR. **(C)** WT and *Bcl3*$^{-/-}$ BMDMs were stimulated with CpG (10nM for 60 mins) and *Fos* and *Nr4a1* mRNA measured by QPCR. Data are presented as the mean -/+ s.e.m and analysed by Students t-Test. *p<0.05; **p<0.01;***p<0.001.

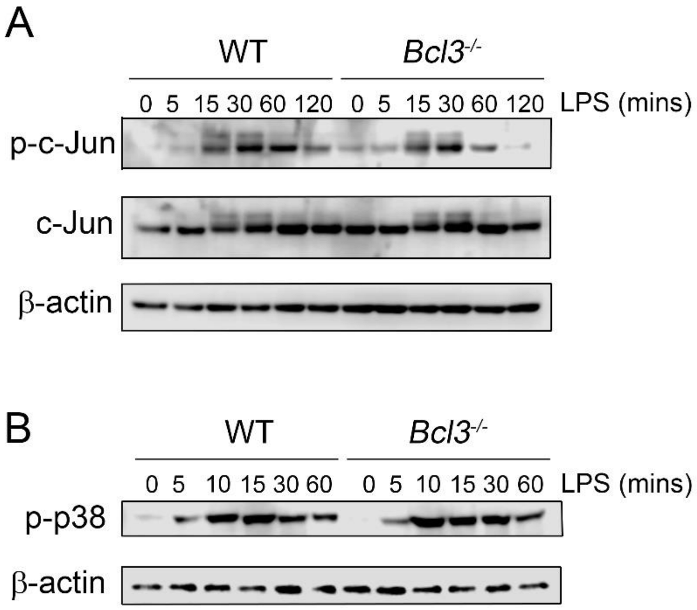

Fig. S2. Equivalent activation of p38 and c-Jun in wild type (WT) and *Bcl3*$^{-/-}$ macrophages. WT and *Bcl3*$^{-/-}$ BMDMs were stimulated with LPS (10ng/ml) for the indicated times, and phosphorylation of c-Jun **(A)** and p38 **(B)** analysed by immunoblot. Data representative of 3 independent experiments.

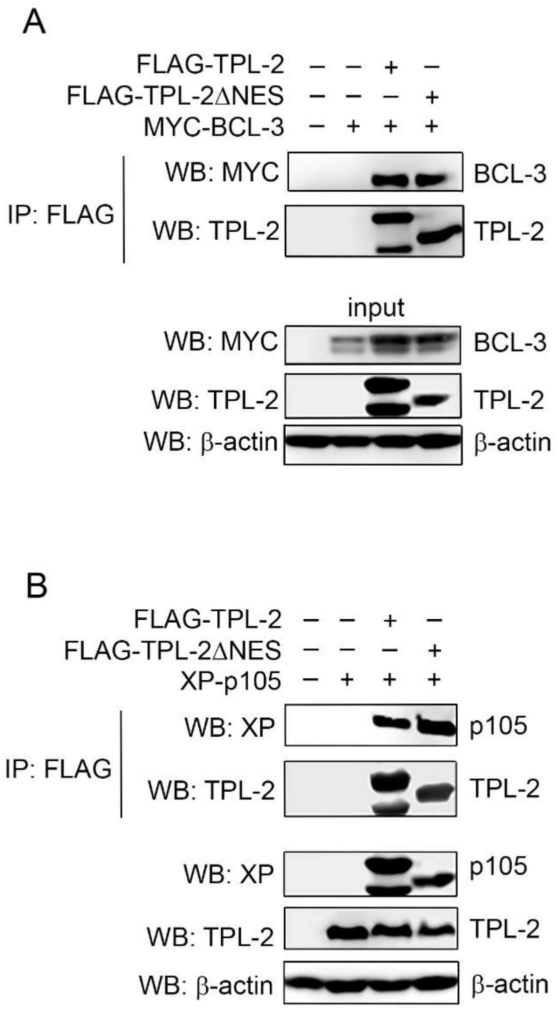

Figure S3. The nuclear export sequence of TPL-2 is not required for interaction with p105 or BCL-3. (A) HEK293T cells were transfected with plasmids encoding FLAG-TPL-2, FLAG-TPL-2ΔNES and MYC-BCL-3 for 24 hours as indicated. TPL-2 and TPL-2ΔNES were immunoprecipitated and interaction with BCL-3 assessed by immunoblot using the antibodies indicated. **(B)** HEK293T cells were transfected with plasmids encoding FLAG-TPL-2, FLAG-TPL-2ΔNES and XP-p105 as indicated. TPL-2 and TPL-2ΔNES were immunoprecipitated and interaction with p105 assessed by immunoblot using the antibodies indicated. All data shown are representative of at least three independent experiments.

CRISPR/Cas9 TPL2 knockout

A. sgRNA: 5’-**TGTCAGATATGGGACCGTGG-3’**

B. **Tpl2-/- Sequence Analysis**

```
Wt              ATGTATCCAGACAGCAATCAAAATGAGGAACGTTCGGAGTCACTGCTTCGGAGTGGCCAG
Tpl2-/-         ATGTATCCAGACAGCAATCAAAATGAGGAACGTTCGGAGTCACTGCTTCGGAGTGGCCAG
                ************************************************************

Wt              GAGGTTCCCTGGCTGTCATCTGTCAGATATGGGACCGTGGAGGATCTGCTTGCATTTGCA
Tpl2-/-         GAGGTTCCCTGGCTGTCATCTG--------------------------------------
                **********************

Wt              AACCATGTCTCCAATATGACAAAGCATTTTTATGGACGTCGACCACAAGAATGTGGAATT
Tpl2-/-         ------------------------------------------------------------


Wt              TTATTAAATATGGTATGTTTCTTCTCATCAGTTGAGTGCTGTGTGCTCTGCTTTGCTGGT
Tpl2-/-         --------------------------------------------------CTTTGCTGGT
                                                                  **********

Wt              AGTGCTTCATTGAATGCACAGTGGCATTGAACTTGGCTTAAGAATGGCTGCCAG-
Tpl2-/-         AGTGCTTCATTGAATGCACAGTGGCATTGAACTTGGCTTAAGAATGGCTGCCAGA
                ******************************************************
```

C. **Translation**

Tpl2-/-

M E Y M S T G S D E K E E I D L L I K H L N V S E V I D I M E N L Y A S E E P G V Y E P
S L M T M Y P D S N Q N E E R S E S L L R S G Q E V P W L S S G N Q S P E W P L P N
R F G C S P C S L E A D I Q E H R L W F R S S G G L W K S I L S P R H E D K E K N G V
Q T D P Y R S V Stop A I G C G N P G L F P A Stop E H C Stop V I W C R P M G R Y C P
S L Y G S R R G R V C S G E T G E L W A H E R I Stop N Y L G D E A H S Q G T Stop F S
A L Q E S D P P Stop Y Stop T Stop Q H C I H V Y K S C F G R F W P E C Stop D D Stop
R C L S S Q G P P G N R D I H E P R G D P M S G P F H K S R H L Q P W S H T H P H A
D R H P T L G E A L P S I S L S L L P I H Y P Q A G T S P G R H R W Stop L Q S R H E G
A D R S C P G E E P Q P P P Q S S R P T E T Stop S P E S P K R G P A T M S E S G L C
P L Stop T E E A A E Q E G T T T S Stop E H C Stop F I M H R Q H R R V Stop S A Q E T
A F P L H Stop P R S S G W L L Q Y C S W A T N P G I R L

Figure S4. CRISPR/Cas9 *Tpl2* knockout in Hoxb8 immortalised murine myeloid progenitor cells. (A) sgRNA sequence. **(B)** Sequencing of *Tpl2* in isolated clone. **(C)** Translation of *Tpl2*$^{-/-}$ sequence in (B).

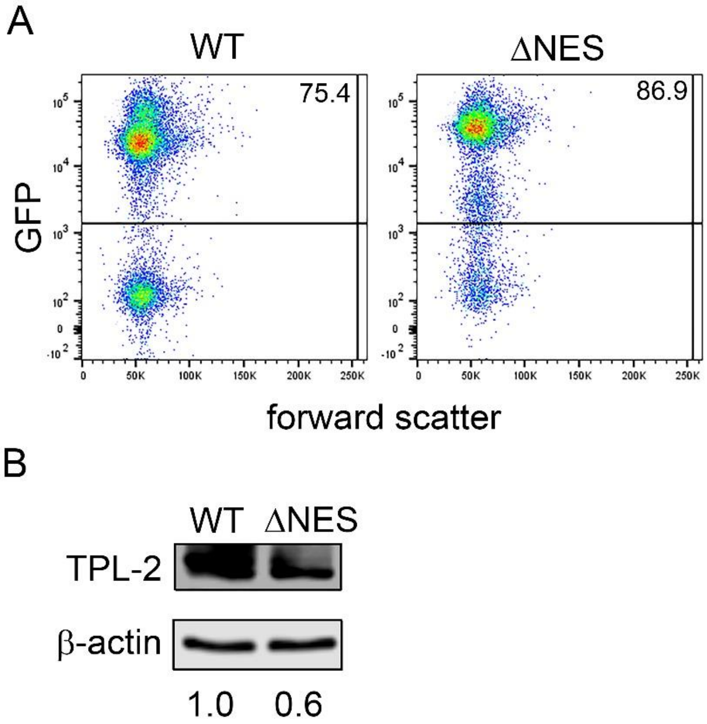

Figure S5. Retroviral mediated expression of TPL-2 and TPL-2ΔNES mutant in Tpl2$^{-/-}$ HoxB8 immortalised murine myeloid progenitor cells. (A) Tpl2$^{-/-}$ cells were retrovirally transduced with TPL-2 (WT) or TPL-2ΔNES mutant (NES). Transduction efficiency was assessed by flow cytometry analysis of GFP co-expressed by the retroviral vector. Numbers indicate percentage of GFP positive cells. **(B)** TPL-2 protein levels were measured in whole cell lysates of cells transduced with TPL-2 (WT) and TPL-2ΔNES mutant (NES). Relative levels of TPL-2 expression normalised to β-actin are shown.

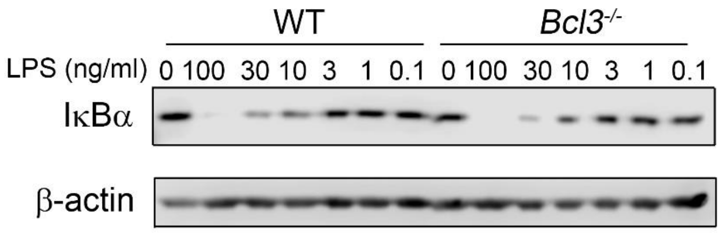

Figure S6. BCL-3 deficiency does not alter activation of NF-κB. WT and *Bcl3*$^{-/-}$ BMDMs were stimulated with the indicated concentrations of LPS and IκBα levels measured by immunoblot. Data are representative of at least 3 independent experiments.

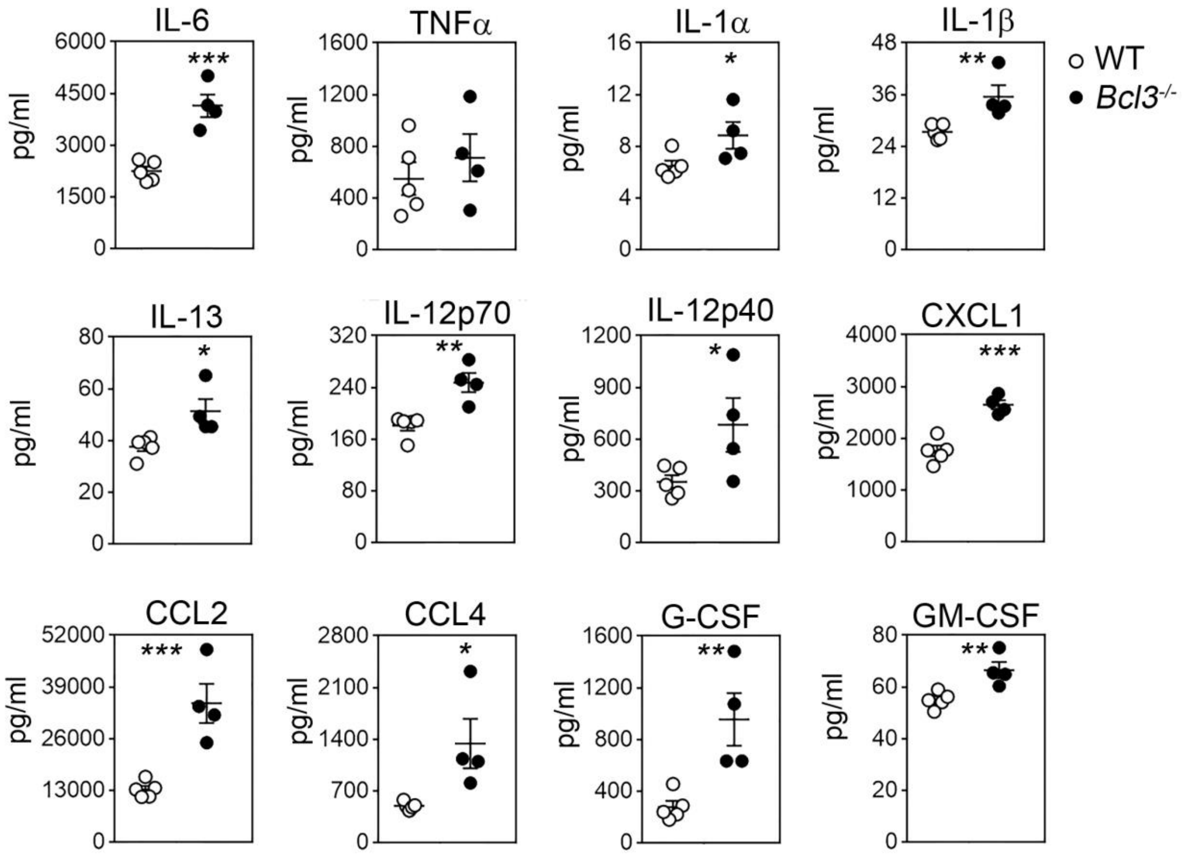

Figure S7. Increased serum cytokine levels in LPS-treated *Bcl3*$^{-/-}$ mice. (A) Serum from WT (n=5) and *Bcl3*$^{-/-}$ (n=4) mice 1 hour after injection of LPS (2.5 µg/mouse *i.p*) was analysed for the indicated factors. Data are analysed by Students t-Test. ****p<0.0001, ***p<0.001, **p<0.01, *p<0.05.
